# Supplementary material for: The Metabolic Changes of Artesunate and Ursolic Acid on Syrian Golden Hamsters Fed with the High-Fat Diet
Source: Molecules. 2020 Mar 18;25(6):1392. doi: 10.3390/molecules25061392 (PMC7144559; doi:10.3390/molecules25061392)
Supplement: Supplementary file 1 [file molecules-25-01392-s001.pdf]

Supporting Information

Table S1. The modeling parameters of all groups.

No.	Model	Type	A	N	R2X(cum)	R2Y(cum)	Q2(cum)	R2	Q2
1	ART-M	PCA-X	2	9	0.5		0.064		
2	ART-M	PLS-DA	3	9	0.531	1	0.904		
3	ART-M	OPLS-DA	1+4+0	9	0.724	1	0.788	1	0.614
4	M-U	PCA-X	2	9	0.475		-0.0512		
5	M-U	PLS-DA	2	9	0.308	0.992	0.239		
6	M-U	OPLS-DA	1+4+0	9	0.709	1	0.49	1	0.786
7	M-combination	PCA-X	3	19	0.442		0.11		
8	M-combination	PLS-DA	2	19	0.19	0.979	0.364		
9	M-combination	OPLS-DA	1+1+0	19	0.19	0.979	0.465	0.932	-0.158

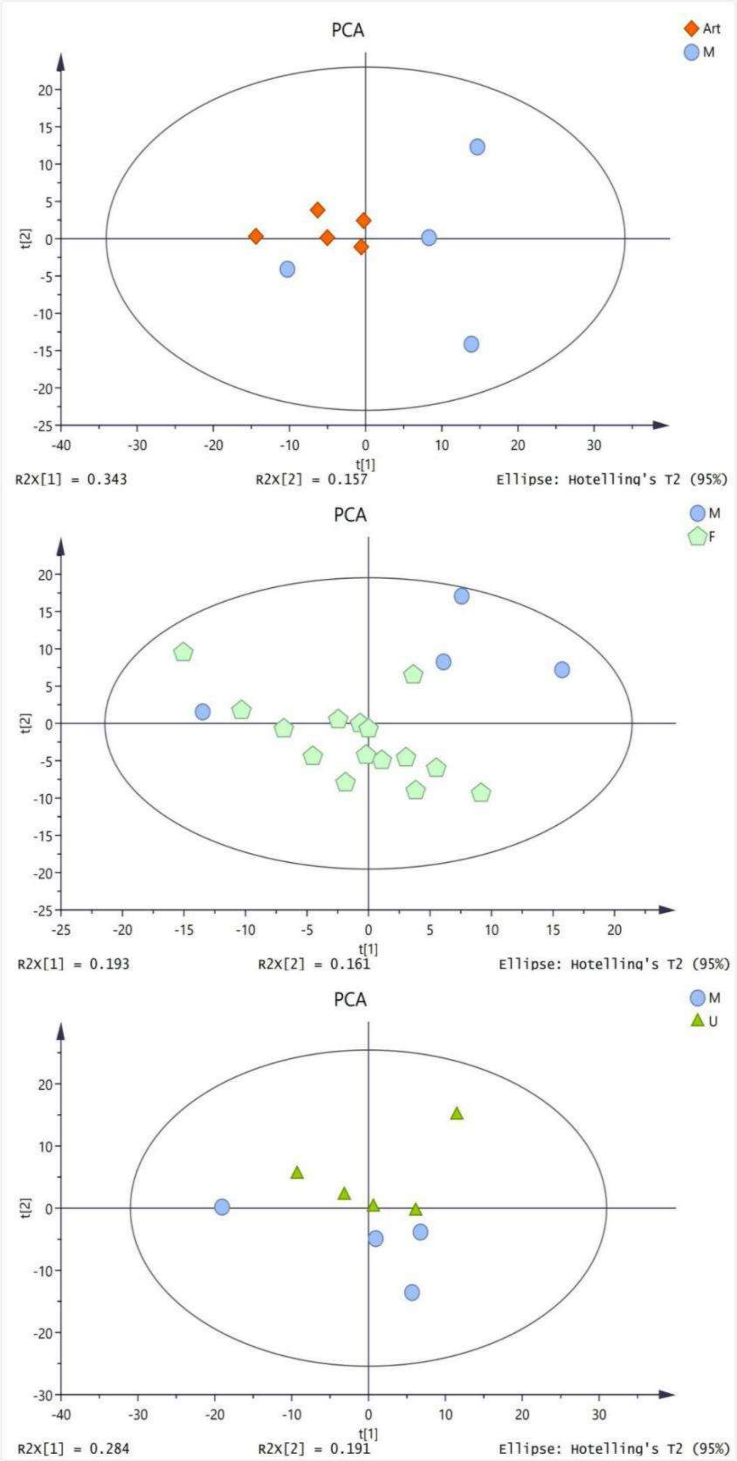

Figure. Unsupervised principal component analysis (PCA) score plots based on the gas chromatography time-of-flight mass spectrometry (GC/TOFMS) data for the model(M) groups vs. artesunate(ART) group (20 mg/kg); ursolic acid(U) group (20 mg/kg) and combination(F) group (artesunate 20 mg/kg + ursolic acid 20 mg/kg).
